# Preclinical evaluation of CD38 chimeric antigen receptor engineered T cells for the treatment of multiple myeloma

**DOI:** 10.1186/2051-1426-3-S2-P13

**Published:** 2015-11-04

**Authors:** Esther Drent, Richard Groen, Willy Noort, Jeroen Lammerts van Bueren, Paul Parren, Jürgen Kuball, Zsolt Sebestyen, Niels van de Donk, Anton Martens, Henk Lokhorst, Tuna Mutis

**Affiliations:** 1 VU University Medical Center Amsterdam, Amsterdam, the Netherlands; 2 Genmab BV, Utrecht, Utrecht, the Netherlands; 3 University of Southern Denmark, Odense, Denmark; 4 Leiden University Medical Center, Leiden, Utrecht, the Netherlands; 5 University Medical Center Utrecht, Utrecht, the Netherlands

## Background

Adoptive transfer of T cells transduced with tumor-reactive Chimeric Antigen Receptors (CARs) is a promising strategy for cancer immunotherapy. The CD38 molecule, with its high and homogenous expression on Multiple Myeloma (MM) cells, appears a suitable target for antibody therapy. Prompted by this, we evaluated the feasibility of targeting MM with CD38-CAR-transduced (CD38-CAR) T cells.

## Methods

We generated three retroviral CAR constructs based on huCD38 antibodies, CD3ζ and 4-1BB signaling domains and transduced them into T cells of healthy donors and MM patients to test the *in vitro* and *in vivo* efficacy.

## Results

Irrespective of the donor, CD38-CAR T cells lost CD38 expression, expanded readily and lysed MM and other malignant cell lines in a cell dose-, and CD38-dependent manner. They also lysed primary malignant cells from acute myeloid leukemia, and multi-drug resistant MM patients. Also in a xenotransplant model, i.v. injected CD38-CAR T cells were effective against MM tumors growing in a human bone marrow-like microenvironment, thus demonstrating their ability to properly migrate and infiltrate into the tumor niche to lyse malignant cells. Although CD38-CAR T cells lysed CD38^+^ monocytes, NK cells, CD34^+^ cells and to a lesser extent CD38^+^ T and B cells, they did not hamper the outgrowth progenitor cells into various myeloid lineages. Furthermore, CD38-CART cells were controllable with a caspase-9-based suicide gene.

## Conclusions

These results signify the potential importance of CD38-CAR T cells as therapeutic tools for CD38^+^ malignancies, including MM, and warrant further safety and efficacy evaluation in appropriate models.

